# Effect of hydroxyapatite on critical-sized defect

**DOI:** 10.1186/s40902-016-0072-2

**Published:** 2016-07-05

**Authors:** Ryoe-Woon Kim, Ji-Hyoung Kim, Seong-Yong Moon

**Affiliations:** 1 Graduate School of Dentistry, Chosun University, Gwangju, South Korea; 2 Department of Oral and Maxillofacial Surgery, School of Dentistry, Chosun University, 309, Pilmun-daero, Dong-gu, 501-759 Gwangju, South Korea

**Keywords:** Bone graft, Hydroxyapatite, Artificial Bone, Xenograft, Bovine bone

## Abstract

**Background:**

Xenologous or synthetic graft materials are commonly used as an alternative for autografts for guided bone regeneration. The purpose of this study was to evaluate effectiveness of carbonate apatite on the critical-size bone defect of rat’s calvarium.

**Methods:**

Thirty-six critical-size defects were created on 18 adult male Sprague-Dawley rat calvaria under general anesthesia. Calvarial bones were grinded with 8 mm in daimeter bilaterally and then filled with (1) no grafts (control, *n* = 10 defects), (2) bovine bone mineral (Bio-Oss®, Geistlich Pharma Ag. Swiss, *n* = 11 defects), and (3) hydroxyapatite (Bongros®, Bio@ Inc., Seongnam, Korea, *n* = 15 defects). At 4 and 8 weeks after surgery, the rats were sacrificed and all samples were processed for histological and histomorphometric analysis.

**Results:**

At 4 weeks after surgery, group 3 (42.90 ± 9.33 %) showed a significant difference (*p* < 0.05) compared to the control (30.50 ± 6.05 %) and group 2 (28.53 ± 8.62 %). At 8 weeks after surgery, group 1 (50.21 ± 6.23 %), group 2 (54.12 ± 10.54 %), and group 3 (50.92 ± 6.05 %) showed no significant difference in the new bone formation.

**Conclusions:**

Bongros®-HA was thought to be the available material for regenerating the new bone formation.

## Background

Various bone graft materials provide osteoconductive matrix to enhance the rate and quality of bone formation in hard tissue-defected region [1]. For the reconstruction of bone defect, autologous bone graft is recommended primarily due to osteogenic, osteoinductive, and osteoconductive properties [2]. However, autograft is often associated with complications at the harvesting site and limited in quantity [3]. Allograft or xenograft is the most commonly used alternative for autogenous harvest, but these materials have a potential risk of disease transmission, rejection, infection, and resorption [4].

Critical-size defect (CSD) is defined as the smallest diameter osseous defect that does not heal spontaneously. Conflicting results have been reported regarding the suitable size of CSD for evaluation of bone regenerative materials in rat calvarial defect model. A full-thickness 8-mm-diameter defect has been suggested as a CSD in rat calvaria [5, 6]. Bilateral calvarial defect has been widely used as a convenient model for testing osteoconductive properties of bone substitutes due to easy handling and low morbidity by reducing the risk of damage in the superior sagittal sinus [7, 8].

Bone apatite is not pure hydroxyapatite (HAp, Ca_10_((PO_4_)_6_OH_2_)). It contains approximately 6 weight % (wt%) of carbonate ions (CO_3_^2−^) as well as other trace elements such as Mg^2+^, Fe^2+^, Na^+^, HPO_4_^2−^, F^−^, and CI^−^ [9]. In terms of its chemical properties, carbonate-containing apatite resembles bone apatite more than pure HAp [10]. HAp has been widely used as a bone substitute because of its excellent biocompatibility and osteoconductive ability. However, its clinical application may be limited because it seems to be not appreciably resorbed [11]. In fact, bioceramics are made of HAp and beta tricalcium phosphate (ß-TCP) and these mixtures have demonstrated bioactivity and osteoconductivity [12].

Past in vivo and in vitro studies have suggested that various autocrine and/or paracrine growth factors in the serum play important roles in wound healing of various tissues, including bone [13]. These growth factors appear to stimulate early phases of wound healing, cell differentiation, and increased primary matrix production, rather than remodeling and maturation processes [14].

This study was performed to evaluate new bone formation in critical-size defects filled with hydroxyapatite and to compare the effect with bovine bone mineral graft material.

## Methods

### Animals

Eighteen adult male Sprague-Dawley rats (each weighing approximately 0.40 kg) were used in this study. The rats were housed in standard cages and were fed under standard laboratory diet. All animal handling and surgical procedures were performed according to the animal care guideline and use of laboratory animals. This experiment was approved by the Chosun University Institutional Animal Care and Use Committee, Gwangju, South Korea (CIACUC2014-A0027).

### Surgical procedure

**Fig. 1:**
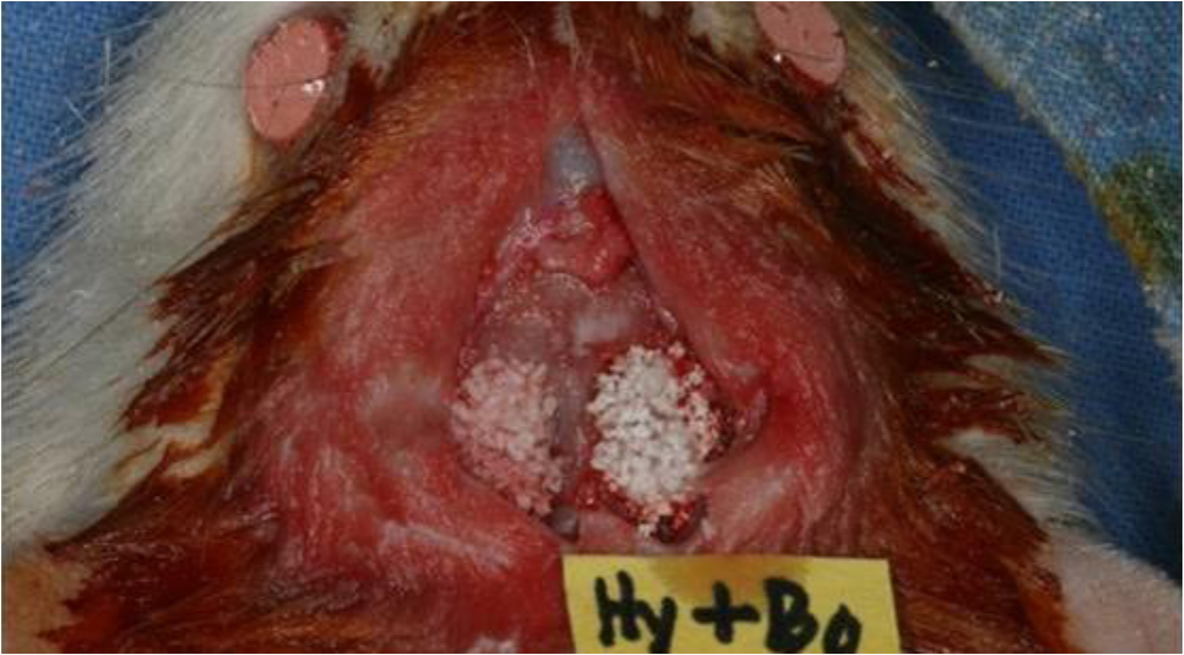
Two defects (diameter ≥8 mm) were created. Bone graft materials were filled in the critical-size defects

General anesthesia was induced by intramuscular injection with 0.5 mg/kg of a combination of Zoletil®50 (tiletamine + zolazepam 1:1; Virbac S.A., Carros, France) and Rompun (xylazine; Bayer Healthcare Korea, Korea) in a ratio of 1:1. After shaving and painting with povidone-iodine, local anesthesia was treated with 2 % lidocaine (Yuhan Co., Seoul, Korea) with 1/100,000 epinephrine. A mid-sagittal incision was performed for exposure of parietal bones. Using a diamond bur, two critical-sized defects (each diameter ≥8 mm) were created in both sides of the parietal bone under normal saline irrigation (Fig. 1). The defects were filled with the following: (1) group 1: no graft as control group (*n* = 10 defects); (2) group 2: Bio-Oss® (Geistlich Pharma Ag., Swiss, *n* = 11 defects); and (3) group 3: Bongros® (Bio@ Inc., Seongnam, Korea, *n* = 15 defects) were used by mixing with saline solution to fill one defect each. The wounds were sutured with Vicryl 4-0 (Ethicon Inc., GA, USA). All the animals received a single intramuscular injection with 0.1 ml/kg of antibiotics (gentamicin, Daesung Microbiological Labs. Co. Ltd., Seoul, Korea) for 3 days.

### Histologic and histomorphometric analysis

#### Histologic analysis

The animals were sacrificed at 4 and 8 weeks after surgery. Histologic samples were harvested including graft sites. The samples were fixed in 10 % neutral-buffered formalin for 3 days and then decalcified in 12.5 % EDTA (Sigma-Aldrich) pH 7.0 for an additional 15 ± 3 days. The samples were serially dehydrated in ethanol in a tissue processor (Shandon Citadel 1000, Thermo Scientific, Franklin, MA, USA) and embedded with paraffin (Leica EG 160). Sections of 5-μm thickness were taken using a microtome (Leica RM 2135). The slides were deparaffinized with xylene and rehydrated with serial concentrations of ethanol.

The slides were stained with hematoxylin-eosin (Sigma-Aldrich) for the use of optical microscope. Using optical microscope, the sections were examined for evaluation of bone formation and integration of the reconstructed areas into the neighboring bone tissue.

#### Histomorphometric analysis

**Fig. 2:**
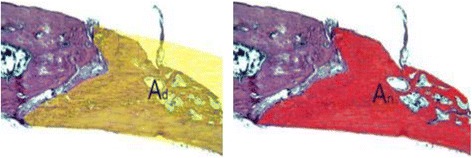
Determination of the bone regeneration. The average fraction of bone regeneration from each group was determined to be the average fraction of bone regeneration: Fraction of bone regeneration = (*A*_n_/*A*_d_) × 100(%) (*A*_d_: the area of the original defect; *A*_n_: the area of the newly formed bone within the defect). First, the assessment of bone healing was performed in each portion and the two portions were summarized by the following formula to evaluate the fraction of bone healing at the whole defect site: The fraction of bone regeneration = (*A*_ln_ + *A*_cn_)/(*A*_ld_ + *A*_cd_) × 100(%) (*A*_ld_ : the original defect at the lateral portion; *A*_cd_ : original defect at the central portion; *A*_ln_: the area of the newly formed bone within the lateral portion defect; *A*_cn_: the area of the newly formed bone within the central portion defect)

Using optical microscope (Primo Star, Carl Zeiss Co., Ltd., Germany) and imaging software (AxioVision 4.7.2), images were obtained for morphology and for analyzing fraction of bone healing. All images were transferred from AxioVision to Adobe Photoshop Elements 7.0 software. Histomorphometric assessment was performed by an individual who was blind to the treatments. The area of the defect and the newly formed bone was measured using Adobe Photoshop Elements 7.0 software. These measurements were used in the following formula to determine the fraction of bone regeneration. The average fraction of bone regeneration from each group was determined to be the average fraction of bone regeneration:

$$ \mathrm{Fraction}\ \mathrm{of}\ \mathrm{bone}\ \mathrm{regeneration} = \left({A}_{\mathrm{n}}/{A}_{\mathrm{d}}\right)\times 100\left(\%\right) $$

where *A*_d_ is the area of the original defect and *A*_n_ is the area of the newly formed bone within the defect site (Fig. 2).

Because bone regeneration was processed from the edge of the defect and towards the center of the defect, two portions (lateral and central portions) of the defect were used. First, the assessment of bone healing was performed for each portion, and then, the two portions were summarized by the following formula to evaluate the fraction of bone healing at the whole defect site:

$$ \mathrm{The}\ \mathrm{fraction}\ \mathrm{of}\ \mathrm{bone}\ \mathrm{regeneration} = \left({A}_{\ln }+{A}_{\mathrm{cn}}\right)/\left({A}_{\mathrm{ld}}+{A}_{\mathrm{cd}}\right) \times 100\ \left(\%\right) $$

where *A*_ld_ is the area of the original defect in the lateral portion, *A*_cd_ is the area of the original defect in the central portion, *A*_ln_ is the area of the newly formed bone within the lateral portion of the defect, and *A*_cn_ is the area of the newly formed bone within the central portion of the defect.

### Statistical analysis

All data were expressed as mean and standard deviation. The new bone formation rate was analyzed via one-way ANOVA and post hoc test.

## Results

After surgery, no visible complication was seen in any rats and wounds.

### Histologic findings

During the process of paraffin embedding, the scaffold composed of bone graft materials was dissolved and the space was represented as empty. And new bone formation was first detected as clusters of globular structures.

**Fig. 3:**
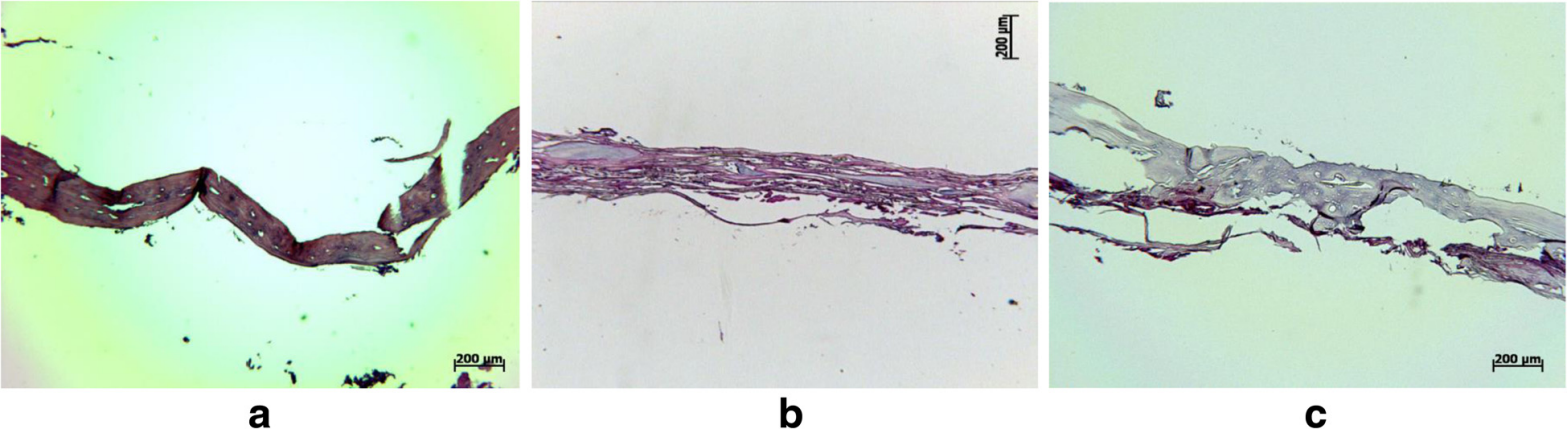
The newly formed bone in the central portion at 4 weeks after treatment: group 1 (**a**), group 2 (**b**), and group 3 (**c**)

**Fig. 4:**
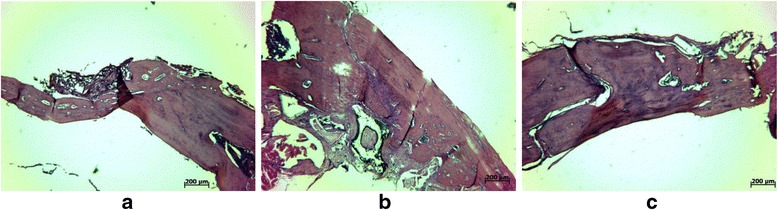
The newly formed bone in the lateral portion at 4 weeks after treatment: group 1 (**a**), group 2 (**b**), and group 3 (**c**)

In the central portion at 4 weeks (Fig. 3), group 1 (control, Fig. 3a) seemed to have less newly formed bone and connective tissue compared to group 2 (Fig. 3b) and group 3 (Fig. 3c). However, half of the defect was filled with the newly formed bone in the lateral portion of group 3(Fig. 4c) at 4 weeks, and the amount of the newly formed bone was similar to that of native bone.

**Fig. 5:**
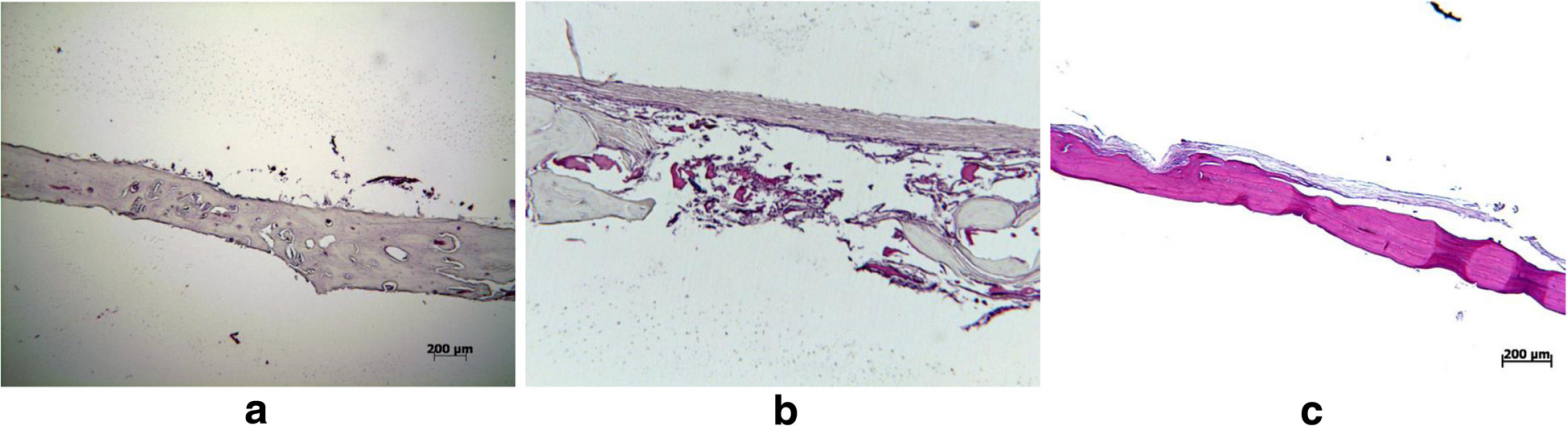
The newly formed bone in the central portion at 8 weeks after treatment: group 1 (**a**), group 2 (**b**), and group 3 (**c**)

**Fig. 6:**
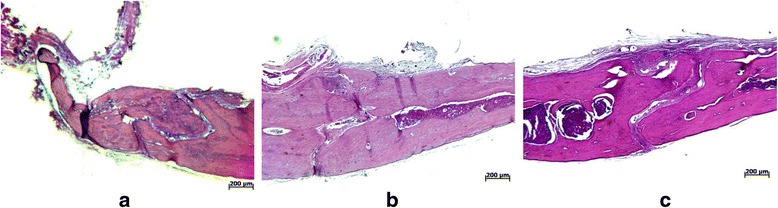
The newly formed bone in the lateral portion at 8 weeks after treatment: group 1 (**a**), group 2 (**b**), and group 3 (**c**)

In the central portion at 8 weeks (Fig. 5), the amount of the newly formed bone was similar to all groups. In the lateral portion at 8 weeks (Fig. 6), the amount of the newly formed bone was similar in all groups except the control group (Fig 6a).

### Histomorphometric analysis

At 4 weeks after surgery, group 3 (42.90 ± 9.33 %) showed a significant difference (*p* < 0.05) compared to the control (30.50 ± 6.05 %) and group 2 (28.53 ± 8.62 %) (Table 1).

**Table 1:** Histomorphometric analysis at 4 weeks after surgery

	Number	New bone formation rate (mean ± SD(%))	Central portion (mean ± SD(%))	Lateral portion (mean ± SD(%))
Group 1 (control)	4	30.50 ± 6.05	21.08 ± 3.45	40.48 ± 15.58
Group 2 (Bio-Oss®)	4	28.53 ± 8.62	13.53 ± 6.25	49.08 ± 16.44
Group 3 (Bongros®)	7	42.90 ± 9.33*	24.56 ± 9.08	63.99 ± 9.14*

*Statistically significant at 0.05 level

At 8 weeks after surgery, group 1 (50.21 ± 6.23 %), group 2 (54.12 ± 10.54 %), and group 3 (50.92 ± 6.05 %) showed no significant difference in new bone formation (Table 2).

**Table 2:** Histomorphometric analysis at 8 weeks after surgery

	Number	New bone formation rate (mean ± SD(%))	Central portion (mean ± SD(%))	Lateral portion (mean ± SD(%))
Group 1 (control)	6	50.21 ± 6.23	28.88 ± 17.29	74.47 ± 12.73
Group 2 (Bio-Oss®)	7	54.12 ± 10.54	25.70 ± 17.23	81.56 ± 7.30
Group 3 (Bongros®)	8	50.92 ± 6.05	36.44 ± 9.52	65.34 ± 11.48

Statistically significant at 0.05 level

In detail, new bone formation was analyzed in two portions—central and lateral. The lateral portion seemed to show higher bone regeneration than the central portion. At 4 weeks in the lateral portion, group 3 (63.99 ± 9.14 %) showed a significantly higher amount of bone formation (*p* < 0.05) compared to other groups. In the central portion at 4 weeks, group 2 and group 3 showed similar new bone formation compared to the control group. At 8 weeks in the lateral portion, all groups showed similar value of new bone formation. At 8 weeks in the central portion, all groups showed similar value of new bone formation.

In summary, group 3 (Bongros) showed significant difference compared to the control (group 1) and group 2 (Bio-Oss) in new bone formation at 4 weeks after surgery, especially the lateral portion of defect. At 8 weeks after surgery, there were no differences among groups 1, 2, and 3.

## Discussion

Critical-size defect (CSD) is defined as the smallest diameter osseous defect that does not heal spontaneously. A full-thickness 8-mm-diameter defect has been suggested as a CSD in rat calvaria [5, 6]. However, several experimental studies have suggested that non-CSD may also be meaningful, in which commercially available bone substitutes showed no osteoconductive properties nor impeded new bone formation in rat calvarial defects [15, 16]. Other studies have reported that 5- or 6-mm-diameter defects also fulfill the requirements of a CSD in rat calvaria [7, 8, 17]. However, Park et al. [18] reported that a 5-mm-diameter calvarial defect is not a CSD in rats because unfilled defects achieved a high degree of new bone formation at 8 weeks. Based on these review of literatures, 8-mm diameter was formed as a CSD in the rat calvaria in this study for strict examination.

HAp has a few disadvantages such as slow absorption rate and low solubility due to low component of carbonate (whereas natural bone has 2.3–8 %/g of carbonate) [19]. Substitution of the functional structures, OH^−^ or PO_4_^−^, of HAp to CO_3_^−^ improves these disadvantages [20, 21].

HAp ceramics have good biocompatibility and osteoinductivity, but no osteogenecity [22]. Various products made by HAp seems to have different osteoinductivities [23], in which one of the reason is porous structures, the degree of interconnection between pores and interconnected pore size [24]. When the interconnected pore size was over 100 μm, osteocytes can differentiate by sufficient nutrition via angiogenesis [24, 25]. However, the optimal size was reported to be 300 μm in literatures, and the bone growth can be inhibited when interconnected pores are too large [26]. Bongros® used in this study has a pore size of 300 μm.

The anorganic bone derived from bovine has reported satisfactory results in various cases of clinical and animal studies for many years, but the range of new bone formation was shown in large variations (5–42 %) [27, 28]. Jensen et al. [27] reported that new bone formation occupied 4 % (2 weeks), 26 % (4 weeks), and 42 %(8 weeks) in the mandibular angle of minipigs. The proportion was lower than that of autogenous bone or tricalcium phosphate (TCP) but was regarded as a favorable result. Kim et al. [29] also reported that new bone formation was more vigorous in the HAp group than in the bovine bone mineral group. However, between the HAp group and the bovine bone mineral group, there is no significant difference of quantity of the newly formed bone in this study.

## Conclusions

The purpose of this study was to evaluate new bone formation in rat calvarial critical-sized bone defects. HAp united bone tissue and provided effective space to make new bone ingrowth, and it was similar to the effect of new bone formation with Bio-Oss. Bongros® was thought to be the available material for regenerating the new bone formation as a scaffold.

### Consent for publication

Written informed consent was obtained from the patient for publication and any accompanying images. A copy of the written consent is available for review by the Editor-in-Chief of this journal.
